# Experiences of using the iCHANGE digital health application in the curriculum for medical students in Thailand

**DOI:** 10.1186/s12909-025-08287-0

**Published:** 2025-11-29

**Authors:** Nida Buawangpong, Kanokporn Pinyopornpanish, Chanchanok Aramrat, Nopakoon Nantsupawat, Suphawita Pliannuom, Nutchar Wiwatkunupakarn, Amalee McCoy, Chaisiri Angkurawaranon, Wichuda Jiraporncharoen

**Affiliations:** 1 Department of Family Medicine, Faculty of Medicine, Chiang Mai University, Chiang Mai, Thailand; 2 Global Health Research Center, Faculty of Medicine, Chiang Mai University, Chiang Mai, Thailand

**Keywords:** Digital health, Application, Telemedicine, Medical education, Medical student

## Abstract

**Background:**

The iCHANGE digital health application was developed to record and monitor six aspects of health behavior, along with laboratory results. It was implemented as part of a curriculum designed to provide medical students with hands-on experience in using digital health for managing multimorbidity. This study aims to explore medical students’ experiences and perceptions regarding the use of a digital health application in the context of multimorbidity care.

**Design:**

Qualitative case study, utilizing semi-structured interviews.

**Setting:**

Maharaj Nakorn Chiang Mai Hospital, a tertiary care university hospital.

**Participants:**

Fourth-year medical students, using purposive sampling.

**Intervention:**

Participants utilized iCHANGE, an application for recording and monitoring health behaviors, laboratory results, communication features, and clinical decision support system (CDSS) functions. Patients recorded their health-associated behavior while medical students provided monitoring, advice, and health education with guidance from a team of health care professionals.

**Methods:**

Using a semi-structured interview guide, focus groups were conducted between August 2023 and May 2024.

**Results:**

Based on six focus groups involving 26 participants, three key themes emerged: (1) digital health as a physician competency for multimorbid care in the digital society, (2) recognition of the challenges associated with digital health interventions, and (3) the role of digital health in the care of patients with multimorbidity.

**Conclusion:**

Incorporating digital health technology into the medical student curriculum may enhance the preparation of students for multimorbidity care and equip them with essential skills to navigate the evolving health care environment.

**Supplementary Information:**

The online version contains supplementary material available at 10.1186/s12909-025-08287-0.

## Introduction

Multimorbidity is the coexistence of multiple chronic conditions in an individual. It affects disease progression and trajectory through disease-disease interaction, drug-disease interaction, and drug-drug interaction [1]. Nearly half of the patients in high-income countries had one or more morbidities, and the prevalence of multimorbidity significantly increased with age [2]. The current overall global prevalence of multimorbidity was recorded as 37.2%, which has increased over the last few decades [3]. Over 20 percent of the Thai population is affected by multimorbidity, with females and older individuals experiencing the highest rates. The prevalence and patterns of multimorbidity are significantly influenced by factors, especially health behaviors [4]. The high prevalence and complexity of multimorbidity requires an increase in interventions to reduce the disease burden [3, 5]. Digital health has emerged as a promising solution to address these challenges, offering new tools and opportunities to transform health care delivery, such as telemedicine and application [6]. Digital health also offers effective tools for medical education in management of multimorbidity [7]. These could help integrate and improve care for the complex health and social needs of multimorbid patients by increasing access to care, enhancing health education, improving health behavior, and extending the scope of health care [8, 9].

Due to continuous growth in digital health, preparing health care professionals for the effective use of digital health knowledge and skill is crucial [10]. By incorporating digital health training into the curriculum, health care professional students can gain the necessary knowledge and skills to use technology effectively [11]. A strategic plan in this area can improve the ability of health care professionals to integrate digital health skills for improving the care of patients with multimorbidities. There is a recognizable need to integrate this technology into the medical student curriculum [10]. A previous literature review revealed that integration of digital health sessions into the medical curriculum improved the knowledge, attitudes, and skills of medical students in the use of digital health such as an adaptability of digital health in clinical settings [12]. However, the implementation of digital health in medical education has predominantly taken place in developed countries and has primarily involved lectures, discussions, or simulations in classrooms [12–15]. A prior strategy for the initiation and implementation of practical digital health in medical training is crucial for effective integration of digital health into medical education [11, 12, 16, 17].

Medical schools need to prepare for the transformation of training methods to ensure students are equipped to use digital health in multimorbidity care [18]. Similar to global reports such as a 2020 European survey showing limited digital health education [15], Thai medical curricula also face the challenge of preparing students with digital health skills. Additionally, a review of digital health training programs in 2021 illustrated that there were few curricula focused on telemedicine and mHealth [14]. In recent years, the Family Medicine curriculum for medical students at Chiang Mai University has incorporated a pilot section of digital health for fourth-year medical students. This component provided medical students with direct experience in using iCHANGE, a digital health application, to support the care of patients with multimorbidity. Therefore, the aim of this study is to understand the experiences and perceptions of medical students regarding the utilization of a digital health application in the context of multimorbidity care.

## Methods

### Setting

The study setting is the Department of Family Medicine at the Faculty of Medicine, Chiang Mai University Hospital, Chiang Mai, Thailand.

### Study population

Fourth-year medical students who had rotated at the Family Medicine Clinic of the Faculty of Medicine, Chiang Mai University, in the 2022 curriculum year.

### Study design

A qualitative case study was conducted. The objective was to explore medical students’ experiences and perceptions regarding the use of a digital health application in the context of multimorbidity care. We conducted semi-structured group interviews with medical students who had been using an application to provide patient care. The focus group interview method is suitable for medical education research [19]. It is more structured due to the group dynamics, allowing for enhanced discussion and interaction between the interviewer and group members [20].

### Sample size

The sample size was determined based on a previous study that explored medical students’ perceptions of digital health education and reported reaching data saturation with 17 participants across four focus groups [21]. In our study, we planned to conduct multiple focus group sessions, each comprising 4 to 8 participants, until data saturation was achieved. Ultimately, six focus group discussions were conducted, involving a total of 26 participants, at which point no new themes were emerging, indicating that data saturation had been reached.

### Family medicine curriculum for fourth-year medical students

The Family Medicine curriculum for fourth-year medical students is designed to provide a comprehensive understanding of primary care principles, emphasizing the importance of a holistic approach to patient care within the context of the family unit. The curriculum consists of both lecture-based sessions and hands-on practice sections. Digital health was implemented as built-in to curriculum by didactic courses with clinical experiences, ensuring participants engage with both theoretical and practical aspects of health care [10].

This practical training implements digital health topics, including mobile medical applications and telehealth, through the iCHANGE application to enhance the care of patients with multimorbidities. Specifically, fourth-year medical students are involved in home visits to patients who require ongoing management of multimorbidity. During these home visits, students have the opportunity to use the iCHANGE application to assess and monitor various aspects of the health associated behaviors for 3 weeks. The integration of a digital health application in this context aims to empower medical students to play an active role in enhancing behavioral change and promoting overall well-being of patients. There was a total of 250 fourth-year medical students per academic year, and all of them had experienced using iCHANGE. The training session for using iCHANGE for fourth-year medical students was a 1-hour session conducted one week before patient visits, covering application functions, data entry, monitoring, and communication tools.

### iCHANGE application

**Fig. 1:**
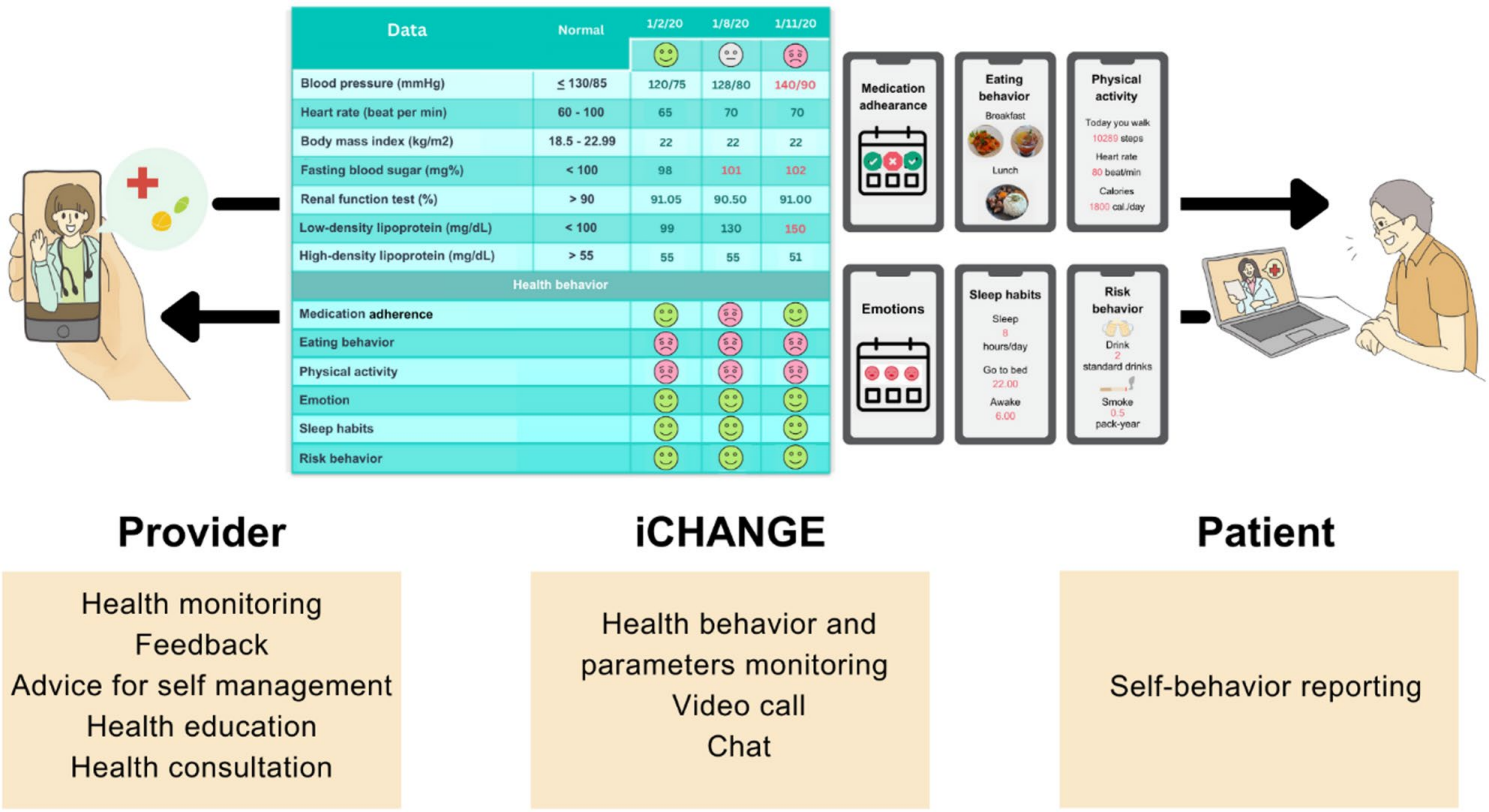
Roles of provider and patient in iCHANGE utilization

iCHANGE was an application for recording and monitoring health behaviors, laboratory results, communication features, and clinical decision support system (CDSS) functions. iCHANGE application could be used by patients, caregivers, and health care providers to record and monitor six aspects of health behavior: food intake, physical activity, medication adherence, sleep, emotional management, and risk behaviors (smoking and alcohol consumption). Patients were provided with a tablet equipped with an installed application to input their health behavior along with an instruction booklet. Patients could input health data into the application, including blood pressure, heart rate, and health behavior (e.g., eating behavior, physical activity). The application would then automatically respond with facial expression icons. A green smiling face icon indicated appropriate behavior, a red frowning face icon indicated behavior that needs adjustment, and a gray neutral face icon indicated no data. This information could be used by both patients and providers as a CDSS for monitoring behavior, disease control, and patient care planning. Additionally, the application allowed for text messages and video calls to communicate with patients. The providers are a team of health care professionals who monitor, provide advice, and give consultations on health behavior and outcomes via the application. Medical students were assigned to take the role of provider under the supervision of health care professionals. Figure 1 illustrates the roles of provider and patient in the utilization of iCHANGE. The iCHANGE application was developed by the Information Technology team of the Global Health and Chronic Conditions Research Center, Chiang Mai University. It is a research-use prototype, not yet released commercially, and therefore does not have a fixed installation size as in app stores. For this study, the application was pre-installed on tablets provided to patients.

### Data collection

Purposive sampling was used. All participants were informed of the research study and provided written consent. Focus group discussions were conducted between August 2023 and May 2024 with permission for audio recording. We conducted semi-structured group interviews with medical students who had been using an application to provide patient care. The focus group interview method is suitable for medical education research [19]. It is more structured due to the group dynamics, allowing for enhanced discussion and interaction between the interviewer and group members [20]. Interview information included baseline characteristics (e.g., age, gender). The interview questions focused on medical students’ experiences and perceptions of using digital health technology for caring for patients with multimorbidity, including (1) their role in patient care, (2) how the application enhances patient health management, (3) how the application contributes to learning about patient care, and (4) suggestions for improvement and expansion. The interview guide is in supplementary file 1. A research assistant, who was not a part of the students’ training, was trained in the interview method and interview questions by WJ and NB. Six groups of fourth-year medical students were interviewed (4 to 6 people per group). Students were grouped mixed. The focus group interviews were conducted within one month after patient care, and each session lasted approximately 40 min in a private and comfortable setting. We used audio recording. Then the interviews were transcribed verbatim. Data collection and analysis were carried out iteratively by the WJ and NB. Researchers listened to the interviews and reviewed notes at the end of each session of data collection to ensure that saturation was achieved. The saturation was identified when there was no new theme emerging from the interview.

### Data analysis

Each transcript was evaluated multiple times to aid familiarization and ensure the accuracy of the data. Descriptive analysis was used to describe participant characteristics including frequency, percentage, mean, and standard deviation (SD). For qualitative analysis, two independent researchers (NB and WJ) conducted an inductive thematic analysis using theoretical frameworks related to digital health competencies [11, 17]. Thematic analysis is a common approach for understanding experiences and perceptions [22]. The preliminary results were then interpreted and discussed with KP, ChA, NN, and CA. Codes were then developed based on patterns in the textual data. The identified codes were compared and discussed for similarities and differences until a consensus was reached on the emergent themes and subthemes. All authors read and contributed to the manuscript. The data analysis was done in NVivo (version 12).

## Results

In this study, each group of 4 to 6 fourth-year medical students was assigned to one patient. A total of 6 patients participated (2 females and 4 males) with a mean age of 62.3 ± 16.7 years. Among the 26 participating students, 18 were male (69%) and 8 were female (31%), with a mean age of 22.2 ± 0.9 years. Three major themes and 7 subthemes emerged from the interviews. The themes were: (1) Digital health as a physician competency for multimorbid care in the digital society, (2) Recognition of the challenges of digital health interventions, and (3) Role of Digital health in multimorbid care. A summary of themes and subthemes is demonstrated in Table 1 Themes and subthemes.

**Table 1:** Themes and subthemes

Theme 1 Digital health as a physician competency for multimorbid care in the digital society
Subtheme 1: Understanding the usefulness of digital health
Subtheme 2: Conducting telemedicine and using CDSS in practice
Theme 2 Recognition of the challenges of digital health interventions
Subtheme 1: Importance of user-centered design and user-friendliness
Subtheme 2: Inequality in using digital health
Subtheme 3: Preparing for the integration of digital health into the service system
Theme 3 Role of digital health in multimorbid care
Subtheme 1: Promoting a patient-centered approach to explore concerns and beliefs
Subtheme 2: Promoting patient self-management

### Theme 1 Digital health as a physician competency for multimorbid care in the digital society

Using the ICHANGE application provided medical students with an opportunity to practice using digital health in patient care. It encouraged them to understand the usefulness of digital health. The majority of students discussed the conducting of telemedicine (video call) and utilization of clinical decision support systems. In addition, they perceived that the use of digital health will become an important aspect of delivery care for multimorbid patients in the future. Therefore, there is a need for physicians to be prepared with the skills and attitudes pertaining to the use of digital health.

### Subtheme 1 Understanding the usefulness of digital health

The benefits of using digital health, such as increasing the access of patients to care without the need for travel, monitoring health behavior, and allowing provider care simultaneously with multiple patients were mentioned. Some medical students perceived that digital health in the form of health applications like iCHANGE has the potential to become a useful tool for patient care in the future, in an era where technology plays an increasingly significant role.

“*It can help reduce travel time and allow for day-to-day follow-up with patients*,* as well as track patient medication intake. It’s a fundamental feature of the app that works quite well.*” (Student 08).

“*I think it helps with tracking patients’ blood pressure when they come to the hospital. Writing it down on paper can easily get lost*,* and relying on memory isn’t always reliable.*” (Student 20).

*“The app includes various lab values*,* which can be viewed from the most recent results to past records. For example*,* we can track lipid levels to see whether they remain stable or have changed over time.”* (Student 03).

“*Having this app can help manage multiple families simultaneously.”* (Student 26).

“*The world is changing with the times*,* and apps make it easy for patients to access care. There are also plenty of platforms on the internet where patients can communicate with doctors. We should have enough doctors who are ready and able to use digital health.*” (Student 26).

### Subtheme 2 Conducting telemedicine and using CDSS in practice

Medical students had practiced providing telemedicine to multimorbid patients under health care team supervision using a mobile health platform as an application. This approach gave medical students the experience of follow-up care, such as monitoring patient blood pressure after adjusting medication and addressing health questions through chat and video calls. iCHANGE has a CDSS that responds to the health data recorded by patients, indicating the level of their health behaviors. This data has helped medical students in making decisions on how to support individual patients in making behavioral changes. They communicated with patients by linking health behavior data with disease management.

*“We used the app to follow up on how the patient is doing after adjusting the medication by reviewing the blood pressure data that the patient inputs every day.*” (Student 13).

*“Patients can ask us health questions anytime via chat*,* avoiding incorrect internet information or unaskable questions. So*,* their doubts can be clarified immediately.*” (Student 24).

*“It is good that the application has face icons. So*,* if the green smile icon is shown*,* it means that the patient has good health behavior*,* and we can encourage the patient to continue the good behavior.”* (Student 09).

### Theme 2 Recognition of the challenges of digital health interventions

Medical students gained different perspectives on the use of digital health in the care for multimorbid patients. Some of these perspectives revolve around the limitations faced by both patients and providers when using the application. Patients may not use the app due to its complex design and functionality. Therefore, user friendliness should be considered. In addition, the issue of equality in access to digital health was addressed such as lack of digital technology skills, physical limitations often associated with elderly individuals, and simply not owning a smartphone. Meanwhile, providers may face an overload of work resulting from an increasing number of cases if the service system is not well-planned. However, they mentioned some ideas of managing these limitations in order to assist patients to use digital health effectively.

### Subtheme 1 Importance of user-friendliness

Medical students recognized the importance of the consideration of patient perspectives when designing an app like iCHANGE. They emphasized prioritizing a user-centered approach to ensure the app is user-friendly, minimizes burdens on the patient, is easy to comprehend, and seamlessly integrates with patient health needs. They recommended the inclusion of user manuals within the application in order to help users navigate the system. Additionally, they proposed developing a user interface that is suitable for older adults.

“*It would be better if patients can contribute to the development of the app together with us. We would like to have a manual available within the iCHANGE app. It would be helpful for patients in case they forget how to use it.*” (Student 07).

“*The most important aspect is its user-friendliness*,* so that patients don’t feel burdened by it. This will encourage patients to gather information more enthusiastically*,* However*,* when it comes to technology and app usage*,* it may make patients feel that it’s too complicated*,* and data might get lost.*” (Student 01).

*“If someone is going to improve the app*,* they should make it comfortable and suitable for elderly users. The text size may need to be larger*,* and the topics should be better organized.*” (Student 01).

### Subtheme 2 Inequality in using digital health

Medical students were aware of the inequality in using digital health. The different levels of access to technology among patients make it challenging for them to use iCHANGE. For instance, patients with limited literacy or education, individuals who are unfamiliar with using technology, elderly patients, and patients who do not own smartphones would have difficulty accessing this digital health service. Furthermore, medical students raised the issue of the need to spend time teaching patients how to use iCHANGE in order to empower them.

“*For the group that is not literate*,* we should study how to make the iCHANGE app suitable and understandable for them.*” (Student 02).

“We *have to be aware that technology seems to be challenging for the elderly already.*” (Student 01).

“*It will be difficult to access to this service for* s*ome patients who used mobile phones with physical buttons instead of smartphones.*” (Student 07).

“*In truth*,* using the iCHANGE app is not difficult*,* but the time allocated for teaching patients how to use the app may not be sufficient. We should strongly recommend that patients actively utilize it.*” (Student 21).

### Subtheme 3 Preparing for the integration of digital health into the service system

Medical students recognized the limitations of digital health. Failing to plan for the implementation of digital health in the care system may negatively impact both providers and patients. It is essential to ensure that service slots and workloads are appropriate. Some students perceive digital health as an additional burden for health care providers when there is insufficient support and resources. If providers cannot actively respond to patients via the application, patients may be more easily lost to follow-up in the system. In addition, patients may feel anxious or stressed from continuous monitoring through the iCHANGE system over an extended period. The service protocol, such as the follow-up time and frequency, needs to be carefully planned.

“*If we have to follow up on every case*,* it would increase the workload for doctors even beyond their working hours. Without a good system*,* it would be an additional burden for doctors.*” (Student 18).

“*Two ways of communication are good. Sometimes*,* when patients ask questions and the medical team doesn’t respond in time*,* it can lead to patients missing out on follow-up within the app.*” (Student 20).

“*It seems ok for patients to be monitored for adjusting health behavior but in the long run*,* they might feel constrained*,* as if someone is constantly watching them. I think there needs to be an awareness regarding the psychological aspect as well as in terms of health outcome”* (Student 04).

### Theme 3 Role of digital health in multimorbid care

Based on the experience of delivering digital health care for multimorbid patients, most medical students reflected on the role of digital health in managing multimorbidity. Firstly, it encourages health care providers to use a patient-centered care approach. The app helps gather behavioral health information, which assists providers in understanding patient concerns and their experience of living with multiple chronic diseases. Secondly, the app promotes patient self-management. By monitoring their health behaviors, patients can learn how to manage their conditions. The health care team can then provide feedback on specific behaviors, helping patients adjust and improve their self-management.

### Subtheme 1 Promoting a patient-centered approach to explore concerns and beliefs

When caring for multimorbid patients, health care teams require to understand not only what diseases the patients have, but also how they live with those diseases. Therefore, their concerns regarding the disease and the contextual meaning of their beliefs should be explored. After reflection, the medical students said that the application helped them understand patients better through observing their behavior. They discussed patients who had expressed concerns that taking medication can lead to kidney failure. Such beliefs can significantly impact medication compliance. The provision of accurate information to patients can help them to understand their own conditions and adopt appropriate behaviors.

“*It’s about bridging the gap between doctors and patients. When we receive pictures of the patient’s meals*,* we gather additional information about their dietary habits and lifestyle*,* enabling us to understand the patient on a deeper level beyond academic content or the disease itself.*” (Student 26).

“*Some patients*,* concerned about taking multiple types of medication*,* believe it can lead to kidney problems. Therefore*,* they might ask questions via text message through the application. It is important to have a conversation to understand why they are not taking their medication and to explain the consequences of non-adherence.*” (Student 26).

### Subtheme 2 Promote patient self-management

Most students reflected that monitoring health behavior is important, as these behaviors are associated with the occurrence of multimorbidity. Digital health can assist in monitoring these aspects, including medication adherence and appropriate dietary choices. Monitoring these behaviors promotes patient self-management and assists in the planning of disease management. Furthermore, medical students believe that giving feedback about patients’ health behaviors helps them understand how to manage their diseases better. This feedback not only boosts patients’ confidence in maintaining good health habits but also helps them correct any unhealthy behaviors in a positive way.

“*There’s the issue of medication adherence*,* especially with elderly patients. Some may forget to take their medication. This application helps patients focus more on this aspect. Its feature also allows patients to take photos and document their meals*,* which they can then send to the doctor daily. What I find particularly beneficial is its encouragement of patient autonomy in managing their nutrition. It’s similar to patients monitoring their daily food intake independently.”* (Student 08).

“*Without the iCHANGE app*,* it would have been difficult to keep such accurate records. For patients with issues like hypertension or diabetes*,* we can also help monitor their food intake to see if it’s too salty or sugary. This allows us to provide feedback on which foods should be reduced or adjusted accordingly.*” (Student 14).

*“I used food photos submitted by the patients. I think this is the most useful feature. I use them to analyze patient’s protein intake*,* and when combined with the lab results recorded in the app*,* it’s really helpful. It supports planning for health promotion as well.”* (Student 5).

*“When patients do something good and the doctor responds in the application with positive feedback*,* it increases the patients’ confidence and encourages them to continue doing it consistently.”* (Student 18).

## Discussion

The use of digital health applications for the care of multimorbid patients enables medical students to learn essential aspects of the digital health area of the patient’s lifestyle. They recognized the use of digital health as a competency for physicians in the digital society. Additionally, students recognized the challenges associated with digital health for helping real practicality in the system. These applications also facilitate the emphasis of key concepts in caring for patients with multiple chronic conditions.

Our participants also recognized digital health as an essential future competency for physicians, particularly in multimorbidity care. This aligns with recent studies showing the importance of enhancing digital health competencies among health professionals and integrating these skills into medical curricula [23, 24]. The integration has been shown to strengthen both skills and attitudes of future physicians [17]. Similar to other studies, which demonstrated positive learner outcomes from the integration of digital health into the medical curriculum, our research reinforces the importance of this educational approach. The participants in our study perceived the usefulness of using digital health in patient care, including providing advice on self-care, using application data for continuous monitoring of chronic disease symptoms, preliminary diagnosis, following up after adjusting medication, and discussing health education with patients and caregivers. A study on digital health intervention in the United States, utilizing the mobile application mHealth for patient education on nutrition and blood pressure measurement, led to improved confidence and skills among medical students in providing lifestyle counseling [23]. Students in our study also appreciated hands-on patient interaction using iCHANGE, echoing findings from a United States telehealth course where practice-based learning improved student competency in using telemedicine [25]. The emphasis our participants placed on building confidence in telemedicine use aligns with a Switzerland educational program that successfully enhanced students’ basic telehealth skills for chronically ill and elderly patients [26]. In addition, the generally positive attitudes our participants expressed toward digital health reflect similar findings from a survey of Chinese medical students, who also viewed digital health as an important component of future medical practice [27].

Medical students observed both the potential and limitations of digital health in caring for multimorbid patients. Learning with real practice helps medical students recognize the challenges of digital health. Digital health services should prioritize the creation of a platform that is user-friendly and easily accessible for both patients and health care providers [28]. The evaluation of patient acceptance of digital health technologies is crucial for providing suitable and appropriate health care [29, 30]. Another concern is equality in regard to digital health interventions [31]. Elderly patients, those with limited literacy or education, individuals unfamiliar with technology, and patients without smartphones may struggle to access these services. To address this, it is crucial to empower patients through training and to ensure the availability of necessary resources [30, 32]. Preparation of the health care system to support digital health in the current technological era is also essential. Effective communication and clear protocols, such as guidelines on how and when to follow up with patients using digital health, can improve understanding and usage among both health care professionals and patients [33, 34]. Medical students reflected that digital health supports multimorbidity care by promoting patient-centered care and encouraging patient self-management. A holistic and patient-centered approach, along with effective care coordination and the use of health technologies, is crucial for successful management of multimorbidity [35]. Digital health provides continuous monitoring, support for personalized treatment plans, and the empowering of patients to actively participate in their health management, making multimorbidity care more patient-centered [36–38]. Providers can use this platform to deliver health information and promote self-management, thereby enhancing the ability of patients to take care of themselves [39]. Patients can also monitor their health and make initial behavioral changes based on their health status and the advice of providers [40]. Therefore, utilization of digital health technology helps medical students gain experience in providing better care for multimorbid patients [41].

Integrating digital health into medical curricula is crucial for both medical students and patients in today’s technology-driven era. To optimize learning, curriculum design should include practice sessions that reflect real-world scenarios, rather than relying solely on lecture-based teaching [42]. Additionally, at the faculty level, it is essential to allocate adequate resources and ensure equitable access to technology [16]. These strategies will help students gain a deeper understanding of digital health and its application, particularly in managing multimorbidity. However, it is important to acknowledge that socio-political, economic, and cultural differences across countries can affect how medical school curricula are delivered, presenting unique challenges in implementation [43].

The strength of this study is that it is one of few providing evidence for the use of digital health intervention as an application for multimorbidity patient care in the curriculum for medical students in Thailand. However, there are still some limitations to this study. Firstly, the duration of medical student rotations in the Department of Family Medicine was only four weeks, allowing the students only three weeks to use the application. Consequently, we could not assess changes in patient health outcomes over this period. Secondly, the focus group interviews were conducted by a research assistant and could have influenced the participants’ responses. However, the research assistant is a person who was trained and experienced in qualitative work interviews. In addition, the research assistant is not involved in the students’ training. Thirdly, the results were gathered solely from a single institute. Some students’ views may not have been fully represented; however, we employed stimulating techniques to encourage participation and gather multiple opinions. Fourthly, we did not evaluate separately the previous experience of using health-related applications, which might influence the experiences of the sample group. Lastly, the results of this study reflect the perspectives of the students rather than objective evaluations of skills and attitudes. Nonetheless, they provide foundational insights and information from learners to enhance provision in the medical curriculum in the future.

## Conclusion

The integration of digital health technology into the medical student curriculum has the potential to improve the preparation of students for multimorbidity care and provide them with the necessary skills to navigate the changing health care environment.

## Supplementary Information

Supplementary Material 1.

## Data Availability

The datasets used and/or analysed during the current study are available from the corresponding author on reasonable request.

## Abbreviations

SD: standard deviation
