# Supplementary material for: Experiences of using the iCHANGE digital health application in the curriculum for medical students in Thailand
Source: BMC Med Educ. 2025 Nov 29;26:32. doi: 10.1186/s12909-025-08287-0 (PMC12781397; doi:10.1186/s12909-025-08287-0)
Supplement: Supplementary file 1 — Supplementary Material 1. [file 12909_2025_8287_MOESM1_ESM.docx]

**Interview Guide for Medical Students**

1. What is your role in patient care through the iCHANGE application?
2. Do you think patient care through the iCHANGE application contributes to improving patients' health? How?
3. In your opinion, how should the iCHANGE application be developed or improved? Why?
4. Do you think the iCHANGE application project has the potential for expansion? How?
5. Do you believe the iCHANGE application project helps enhance your learning in patient care? How?
